# Excellent short-term results of dome-shaped high tibial osteotomy combined with all-inside anterior cruciate ligament reconstruction

**DOI:** 10.1186/s40634-023-00632-w

**Published:** 2023-07-10

**Authors:** Toshiaki Takahashi, Seiji Watanabe, Masanori Hino, Haruhiko Takeda, Toshio Ito

**Affiliations:** 1 Department of Sports and Health Science, Faculty of Collaborating Regional Innovation, Ehime University, 3 Bunkyo-Cho, Matsuyama, Ehime 790-8577 Japan; 2 Department of Orthopaedic Surgery, Watanabe Hospital, Matsuyama, Ehime 791-0054 Japan; 3 Department of Orthopaedic Surgery, Saijo Central Hospital, Saijo, Ehime 793-0027 Japan; 4 Department of Orthopaedic Surgery, Murakami Memorial Hospital, Saijo, Ehime 793-0030 Japan

**Keywords:** Dome-shaped HTO, Posterior tibial slope, ACL reconstruction, All-inside

## Abstract

**Purpose:**

This study aimed to evaluate short-term outcomes at least 2 years after dome-shaped high tibial osteotomy (HTO) combined with all-inside anterior cruciate ligament reconstruction (ACL) in patients with persistent ACL insufficiency accompanied by pain due to varus deformity.

**Methods:**

The study enrolled 19 knees of 18 patients. The mean age was 58.4 ± 13.4 years and the mean postoperative follow-up period was 31.4 ± 6.6 months (24–49 months). JOA(Japanese Orthopaedic Association)-OA(osteoarthritis) score, Lysholm score, radiographic outcomes such as femoro-tibia angle (FTA) in a standing position, side-to-side difference in KT-1000 measurements were evaluated at pre op. and post operative final follow up. And arthroscopic evaluation was evaluated at the time of the HTO plate-removal procedure.

**Results:**

Before surgery, the mean JOA-OA score was 65.0 ± 13.5, the mean Lysholm score was 47.2 ± 16.2, the mean femoro-tibia angle (FTA) in a standing position was 183.8 ± 3.4° (range;180–190°), and the mean side-to-side difference in KT-1000 measurements was 4.1 ± 1.3 mm. After surgery, the mean JOA-OA score, Lysholm score, and side-to-side difference in KT-1000 measurements improved to 93.1 ± 6.0 (*P* < 0.00001), 94.2 ± 5.9 (*P* < 0.00001), and -0.2 ± 0.8 mm (*P* < 0.00001), respectively. The mean FTA decreased to 168.0 ± 3.3 (*P* < 0.00001), and the mean posterior tibial slope angle decreased to 5.0 ± 3.6° from 6.9 ± 2.6° preoperatively (*P* = 0.024). Arthroscopic evaluation during the HTO plate-removal procedure of 17 knees were performed at a mean of 16 months after the surgery. The reconstructed ACL graft in 13 knees were successful, a cyclops lesion in one knee, and looseness of the graft in three knees.

**Conclusions:**

Dome-shaped HTO allows for a relatively high degree of varus correction and decreases the steep posterior tibial slope that causes excessive load on the ACL. Therefore, its use in combination with ACL reconstruction seems to be effective.

## Introduction

Osteoarthritis of the knee is sometimes complicated by symptomatic anterior cruciate ligament (ACL) deficiencies [33]. Regardless of the cause, these patients typically present with varus deformities of the knee [14]. Common problems with daily living include medial knee pain, excessive varus–valgus instability when changing direction, and difficulty using stairs [18].

For patients with ACL deficiencies and large varus deformities and significant osteoarthritic changes of the patellofemoral joint, total knee arthroplasty (TKA) is the primary treatment option [9]. However, for patients aged 70 years or younger who have mild patellofemoral lesions and who wish to engage in full sporting activities or live a floor-sitting lifestyle (common in Asian and other regions), joint-sparing procedures involving high tibial osteotomy (HTO) and ACL reconstruction can be a viable choice because TKA patients may not do some sports activity and kneeling [11, 22]. There are different HTO techniques, such as open-wedge [12], closed-wedge [7], and dome-shaped osteotomy [21, 29]. Individual techniques differ in rehabilitation protocols (e.g., time to full weight-bearing status), postoperative tibial length and posterior tibial slope, and time to bone union.

In this series, dome-shaped HTO with medial plate fixation and all-inside transfemoral ACL reconstruction were performed simultaneously due to obtain the high activities and pain relief.

HTO surgery alone does not provide anteroposterior stability of the knee for the patients of osteoarthritis of the knee with ACL deficiency. In contrast, in the combination of the opening wedge HTO and ACL reconstruction, grafted ACL does not work well postoperatively [25]. Opening Wedge HTO is also known to aggravate the posterior slope of the tibia, which increases ACL tension and is problematic in ACL insufficiency cases.

The purpose of this study is to evaluate the short-term results of the combination surgery of dome-shaped HTO and transfemoral all-inside ACL reconstruction.

## Materials and methods

A total of 19 knees of 18 patients (nine men and nine females) with painful varus deformities and persistent instability due to ACL deficiency were included. Their mean age was 58.4 ± 13.4 years (range 41–70 years).

Patient selection: This procedure is indicated for patients under the age of 70 year-old who have persistent pain due to varus deformity and obvious anteroposterior instability of the knee due to ACL insufficiency.

### Inclusion criteria

1. In the femorotibial joint, there is distinct medial articular cartilage degeneration, and lateral articular cartilage degeneration is not marked.
2. Shows varus deformity of the lower extremity (in principle, standing FTA 180° to 190°).
3. Shows anteroposterior instability of the knee, ACL insufficiency shadow on MRI, positive pivot shift test, side-to-side difference of 3 mm or more on KT-1000.
4. Desire for heavy labor or high activity after operation.
5. Consent to rehabilitation plan such as postoperative weight bearing period.

### Exclusion criteria

1. Extensive lateral articular cartilage degeneration of the femorotibial joint.
2. Patients after ACL reconstruction.
3. Desire full-weight bearing walking within 3 weeks after operation.
4. Patients who wish to have an artificial knee joint.
  Arthroscopic assessment of the status of the grafted ligament at the time of plate removal is quantitative and tension of the grafted ligament. The tension was checked by using a probe and pushing the ligament from the front for the anterior fibers, or pulling it forward from the rear for the posterior fibers.

### Surgical procedure

**Fig. 1:**
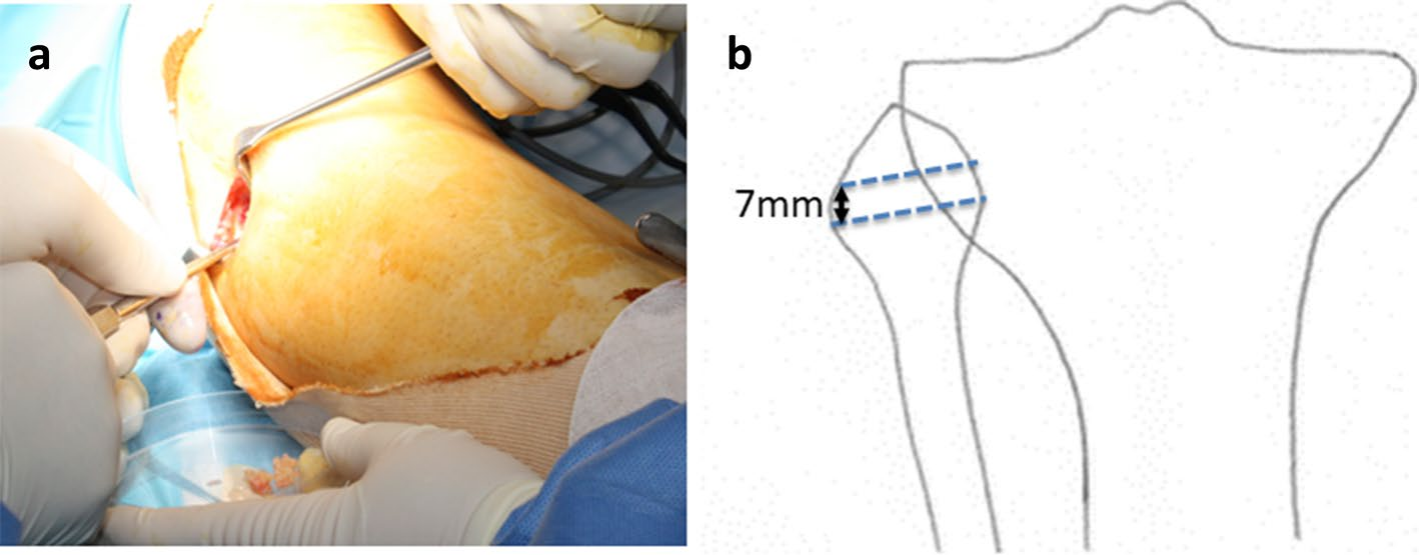
**a** A skin incision of approximately 3 cm is made anterior to the fibular neck and subcutaneously expanded to expose the fibular neck. **b** A distal 15 mm segment of the fibular head was excised with 7 mm height. After drilling with a Kirschner steel wire from the anterior side alone, fibular bone resection was performed with a chisel to excise the anterior cortex and cancellous bone

In the HTO procedure, a fibular bone resection was performed at a site of proximal to the fibular neck with the width of 7 mm (Fig. 1a, b). After drilling with a Kirschner steel wire from the anterior side alone, fibular bone resection was performed with a chisel to excise the anterior cortex and cancellous bone. The biceps tendon and lateral collateral ligament of the knee attached to the lateral side of the fibula are untouched. When varus correction is performed, the space of the bone resection is filled and bone union occurs.

**Fig. 2:**
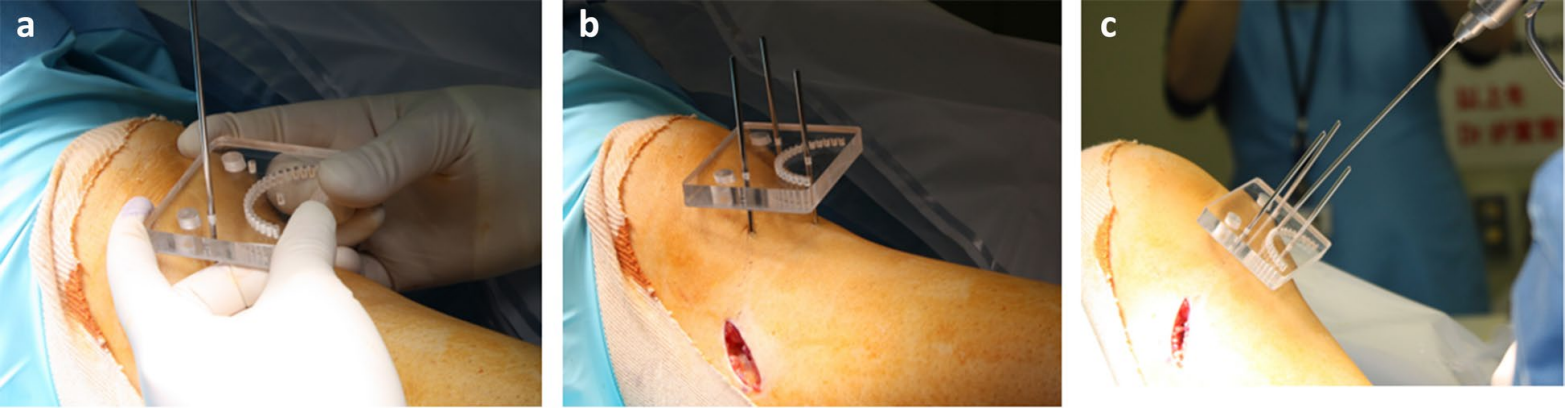
**a** A custom-designed drill guide plate with a series of holes (*n* = 20–30) along a curved line was placed on the tibial skin surface under a fluoroscopic control. **b** A custom-designed drill guide plate was fixed with a Kirschner steel wires of 2.4 mm diameter under fluoroscopic control in the extension of the knee. b The drill guide plate was fixed firmly with three Kirschner steel wires. **c** A Kirschner steel wire of 2.4 mm- diameter was used to create holes percutaneously into the tibia along the curve with 120° of flexion of the knee. Drilling was performed with the Kirschner wire from anterior to posterior cortex of the tibia at low rotation speed for the reaming

**Fig. 3:**
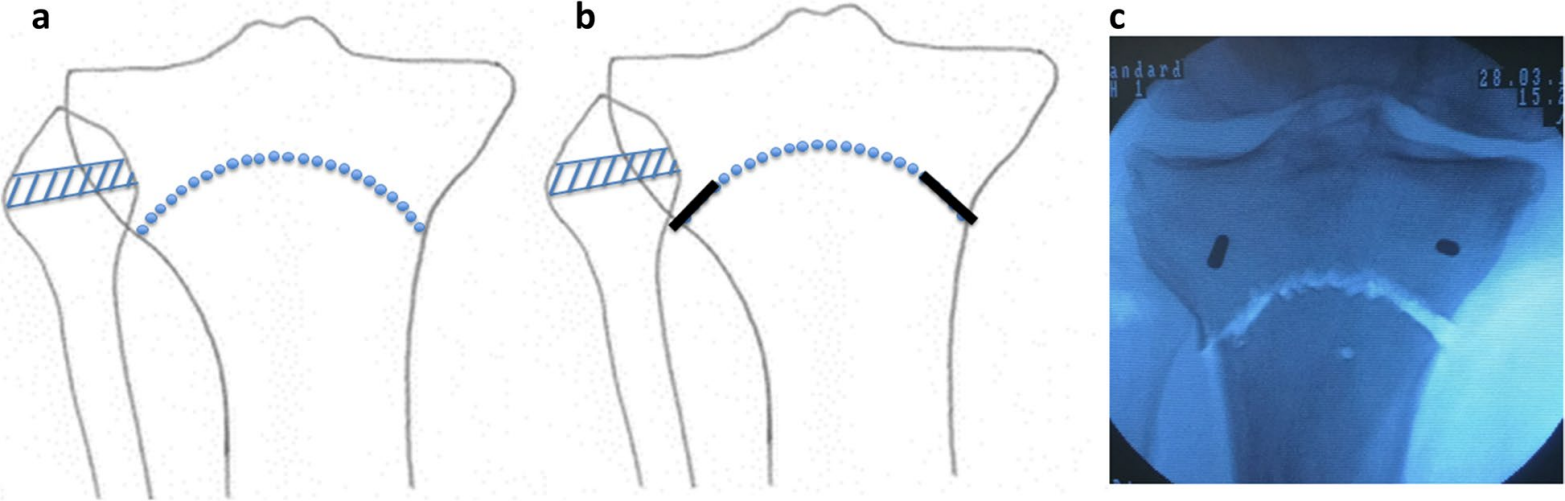
**a** Schema of percutaneous drilling using Kirschner steel wire and resection of fibular neck of a width of 7 mm. **b** A bone chisel (black solid line) was applied over a 1-cm incision made along the curve to cut the medial and lateral cortex of the tibia. **c** A completion of dome shaped osteotomy was confirmed by a fluoroscopy according to apply the varus and valgus stress of the knee

A custom-designed drill guide plate with a series of holes (*n* = 20–30) along a curved line was placed on the tibial skin surface under a fluoroscopic control [29] (Fig. 2a and b). A Kirschner wire of 2.4-mm-diameter was used to create holes percutaneously into the tibia along the curve with 120 degrees of flexion of the knee (Fig. 2c). Drilling was performed with the Kirschner wire from anterior to posterior cortex of the tibia at low rotation speed for the reaming (Fig. 3a). With the knee at approximately 120° of flexion and the popliteal artery relaxed, a kirschner wire is marked usually anteriorly, to a length of 45 mm and drilled at low speed. When drilling from the front, pay attention to the sensation of perforating the posterior cortex, especially at the 10 mm lateral portion from the center of the tibia, and immediately stop turning once the perforation occurs. In addition, even if the posterior cortex is not pierced at that site, the dome-shaped osteotomy can be completed by applying manual stress, so it should not be forced forward.

**Fig. 4:**
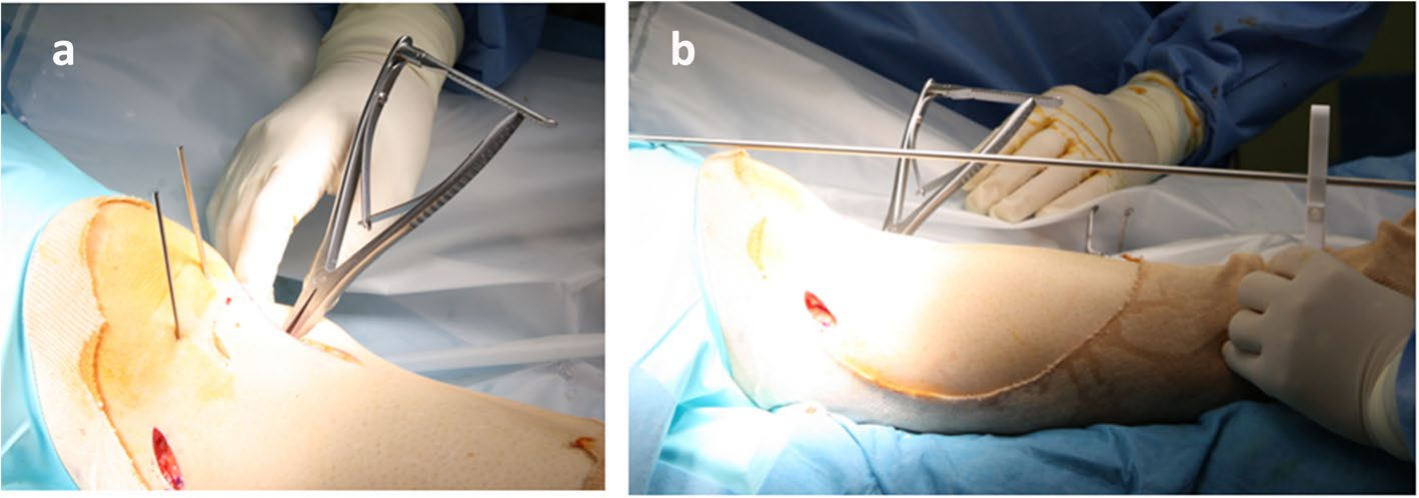
**a** A spreader was inserted into medial gap and performed the adequate correction. **b** An extramedullary rod to correct the alignment was set from the center of the femoral head to the center of ankle joint. An adequate varus correction was performed as an extramedullary rod pass through the lateral thirds of the tibial plateau. The gap width (mm) was measured by a spreader

While applying manual force to the distal part of the tibia in the medial direction, a bone chisel was applied over a 1-cm incision made along the curve to cut the medial and lateral cortex of the tibia (Fig. 3b). Then, a completion of dome shaped osteotomy was confirmed by a fluoroscopy (Fig. 3c). A spreader was inserted into medial gap and performed the adequate correction (Fig. 4a). An extramedullary rod to correct the alignment was set from the center of the femoral head to the center of ankle joint (Fig. 4b). An adequate varus correction was performed as an extramedullary rod pass through the lateral thirds of the tibial plateau. The gap width (mm) was measured by a spreader.

**Fig. 5:**
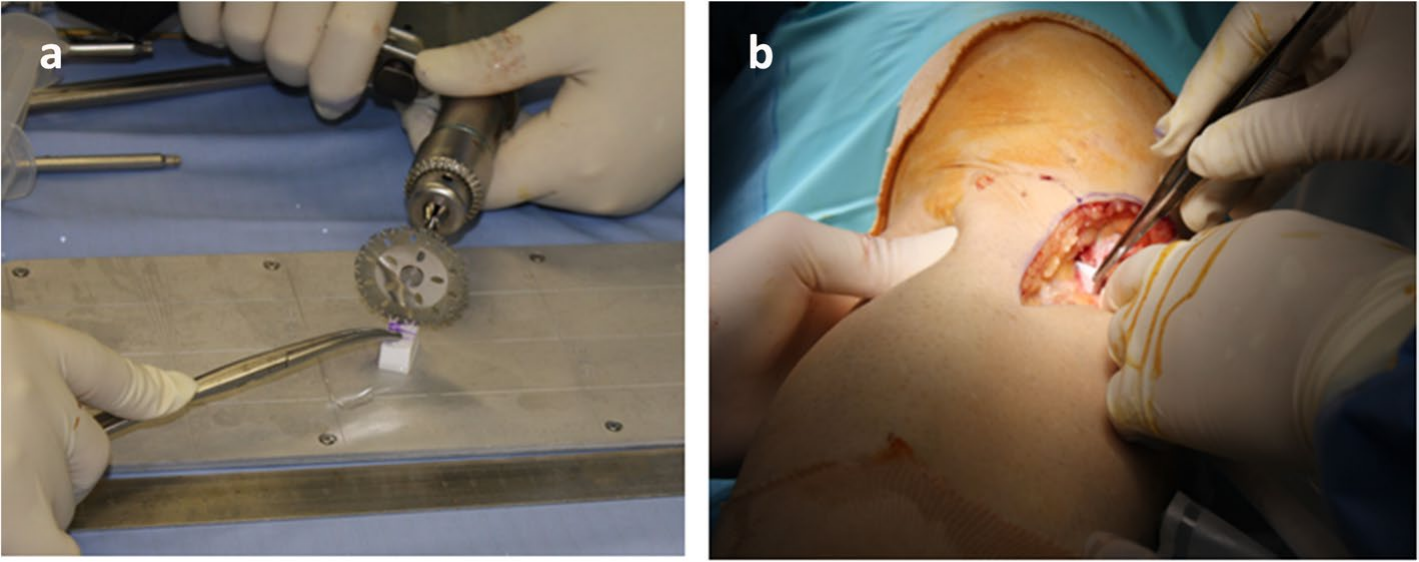
**a** Two trapezoid-shaped synthetic hydroxyapatite bone graft blocks (Osferion®, Olympus Terumo Biomaterials, Japan) (height:10 mm, top:3-5 mm, buttom:7-10 mm), were made according to the gap width. **b** Two trapezoid-shaped synthetic hydroxyapatite bone graft blocks were inserted into a medial and a postero-medial gap

After a satisfactory femorotibial alignment was achieved, two trapezoid-shaped synthetic hydroxyapatite bone graft blocks (Osferion®, Olympus Terumo Biomaterials, Japan) (height:10 mm, top:3-5 mm, buttom:7-10 mm), were made according to the gap width (Fig. 5a). Two trapezoid-shaped synthetic hydroxyapatite bone graft blocks were inserted into a medial and a postero-medial gap (Fig. 5b).

**Fig. 6:**
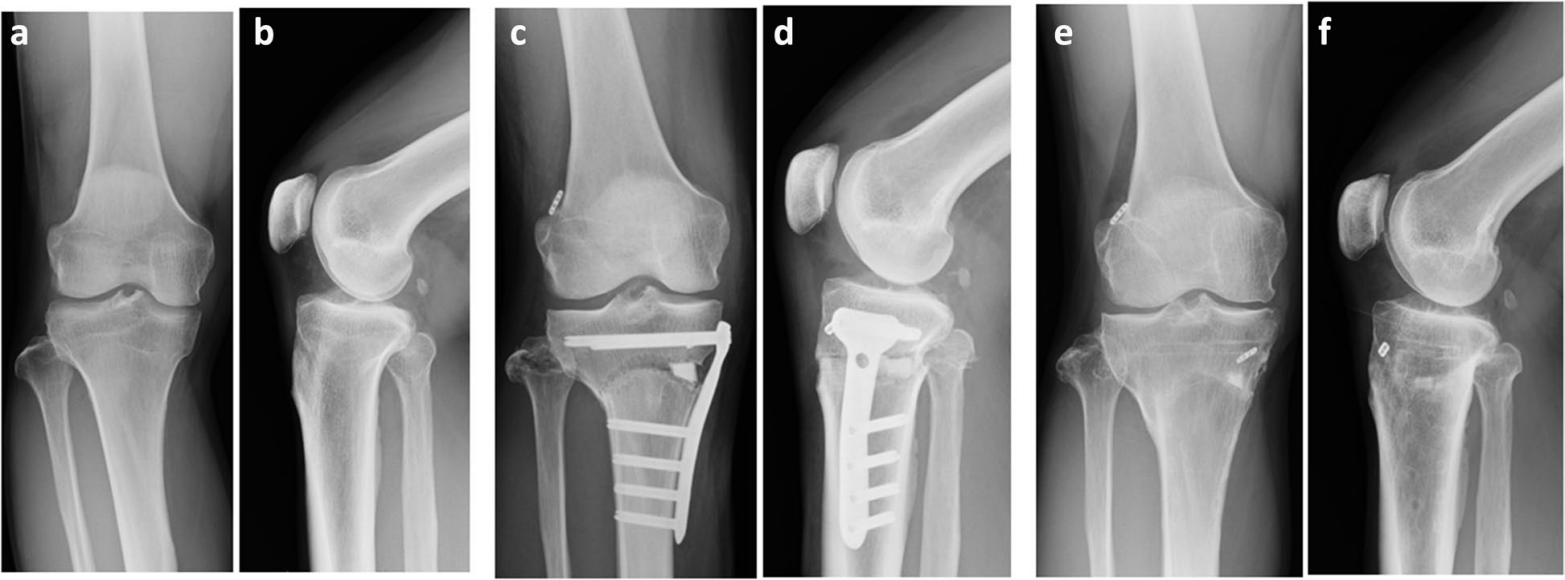
**a** Dome-shaped high tibial osteotomy combined with all-inside anterior cruciate ligament reconstruction using semitendinosus tendon simultaneously at pre and post operative radiography. a Standing antero-posterior view, **b** Lateral view of pre operation. b Standing antero-posterior view, **c** Lateral view of postoperative 3 weeks,. **d** Standing antero-posterior view, **e** Lateral view of postoperative 3 years

The medial aspect of the tibia was fixed with a T‐shaped locking plate (TriS Medial HTO Plate System®, Olympus Terumo Biomaterials, Japan) (Fig. 6c, d).

**Fig. 7:**
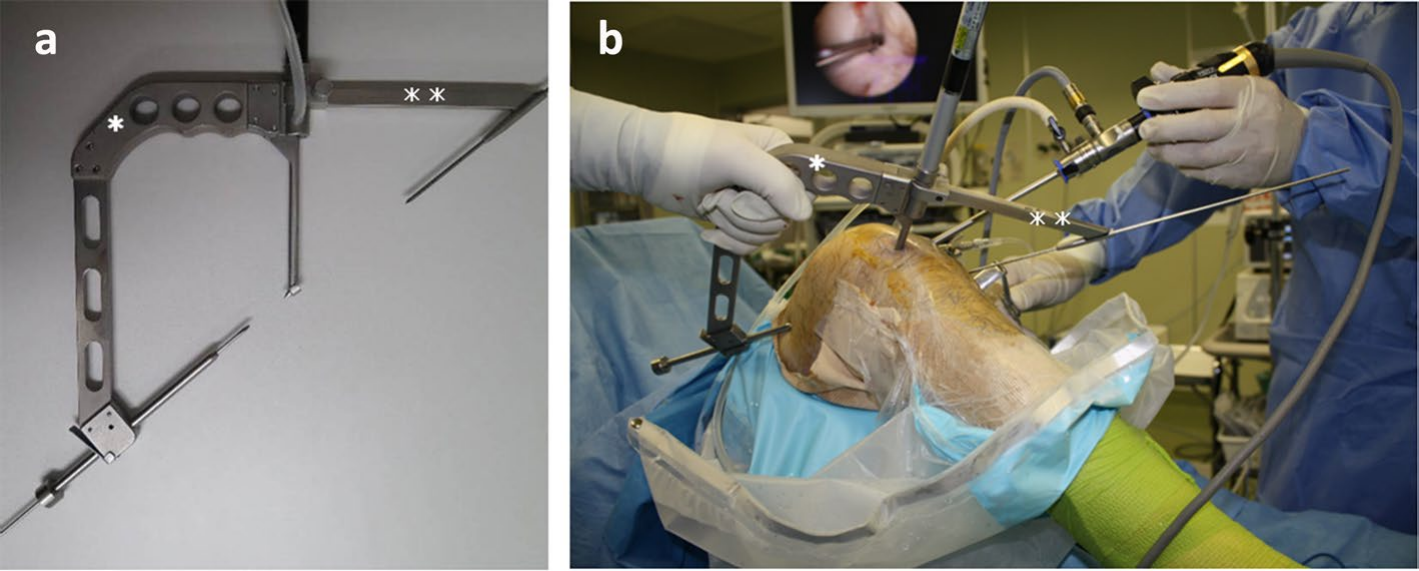
**a** Transfemoral drilling guide (*) and Tibial tunnel guide (**) were used in order to decide the location of the femoral tunnel. A tibial tunnel guide (Hole-in-one guide) is used to check the anticipated location of the tibial tunnel exit on the anterior surface of the tibia. **b** Transfemoral drilling guide (*) and Tibial tunnel guide (**) were set at right knee of 90° flexion and the largest internal rotation angle possible for the transfemoral approach in the dependent method

In the ACL reconstruction, single-bundle quadrupled hamstring (semitendinosus) tendon grafts were used [2]. Transfemoral drilling guide (*) and Tibial tunnel guide (**) were used in order to decide the location of the femoral tunnel (Fig. 7a) [31]. A tibial tunnel guide (Hole-in-one guide) is used to check the anticipated location of the tibial tunnel exit on the anterior surface of the tibia. Transfemoral drilling guide (*) and Tibial tunnel guide (**) were set at right knee of 90° flexion and the largest internal rotation angle possible for the transfemoral approach in a dependent method (Fig. 7b).

**Fig. 8:**
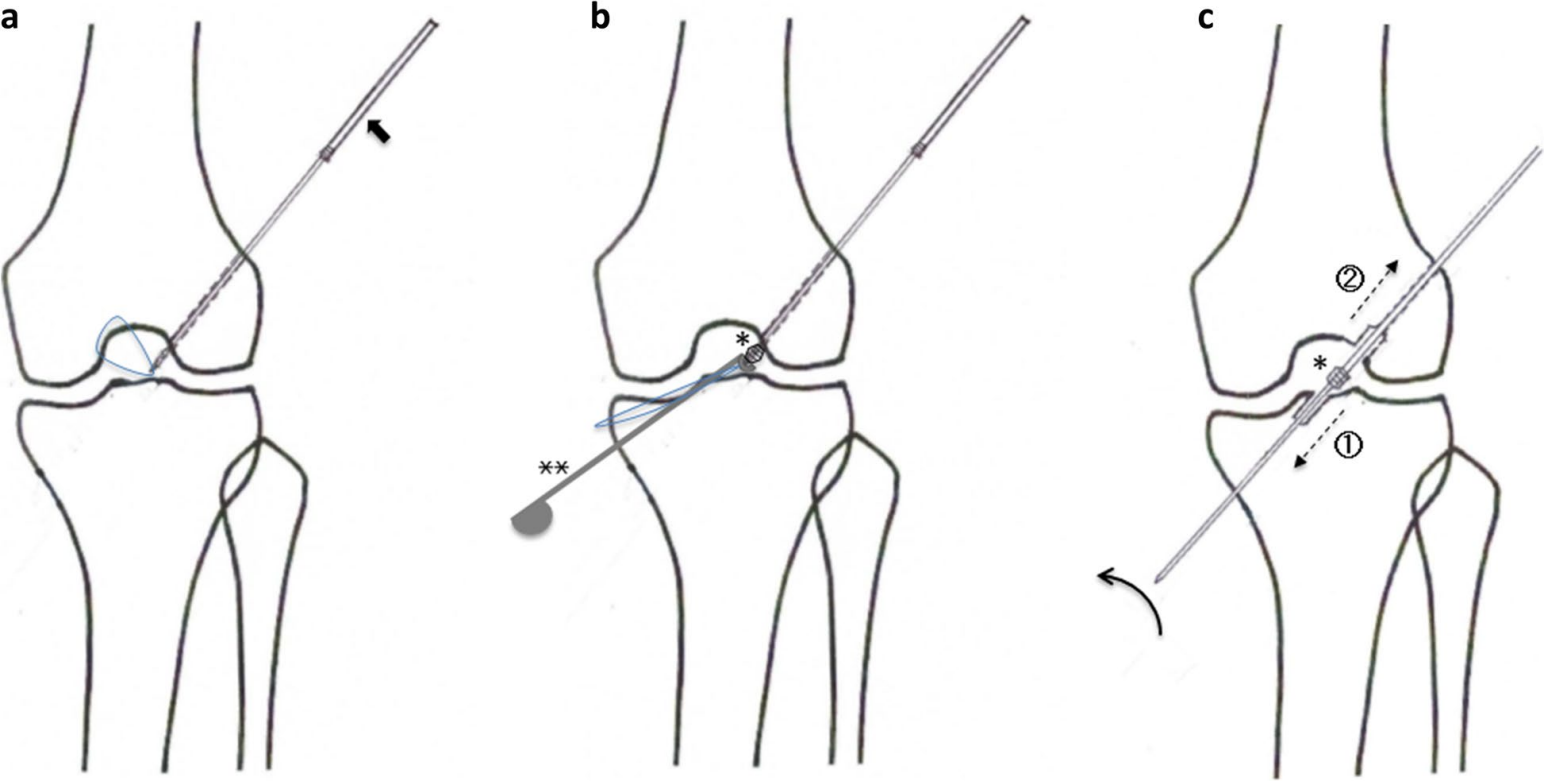
**a** A 2.4 mm-diameter Kirschner steel wire is driven through the targeted femoral ACL footprint site into the intraarticular space. Then, the femoral tunnel diameter is enlarged by applying a 4.5 mm-diameter. A special drill guide pin (Black arrow) which has an apertured tip with 2–0 nylon loop was inserted to intraarticular portion in order to deliver the reamer bit inside the joint. Black arrow: A special drill guide pin. **b** The nylon loop is extracted at outside of the medial portal. A guiding nylon thread is passed through the reamer bit opening (*) and is used to deliver the reamer bit into the intraarticular space using a reamer pusher (**) and passes the drill guide pin through the reamer bit opening. *: The reamer bit opening, **: A reamer pusher. **c** Transfemoral drilling of both tibial and femoral sockets was performed in an all-inside manner using a custom-designed drill guide pin–reamer bit system. The drill pin and reamer are fixed at the inside of the knee joint, and the drill pin was rotated clockwise (curved arrow) and advanced to create the tibial socket (①) and then retracted to create the femoral socket (②) [Takahashi 2023]

2.4 mm-diameter Kirschner steel wire is driven through the targeted femoral ACL footprint site into the intraarticular space. Then, the femoral tunnel diameter is enlarged by applying a 4.5 mm-diameter. Special drill guide pin which has an apertured tip with nylon loop was inserted to intraarticular portion in order to deliver the reamer bit inside the joint (Fig. 8a).

Transfemoral drilling of both tibial and femoral sockets was performed in an all-inside manner using a custom-designed drill guide pin–reamer bit system (Fig. 8b). The drill pin and reamer are fixed at the inside of the knee joint, and the drill pin is rotated clockwise and advanced to create the tibial socket and retracted to create the femoral socket (Fig. 8c) [31].

A hole-in-one guide with the transfemoral drill guide is used, so the anterior tibial exit is visible. Therefore, the tibial bone tunnel can be prevented from contacting the HTO plate. Also, when the Tris plate® is placed from the medial side, the proximal screw faces the posterior side of the tibia, so that the proper bone hole on the tibia side is positioned in front of the screw, so there is no interference between them. In addition, the length of the tibial bone tunnel is less than 30 mm, and it does not interfere with the screw. The graft construct was fixed with an endobutton (ENDOBUTTON CL BTB®, Smith & Nephew, Andover, MA) on the femoral side, and with a fixation by an endobutton alone on the tibial side [31] (Fig. 6e, f).

Immediately after surgery, the patient’s knee is kept at approximately 20° of flexion by a knee brace in order to reduce the tension of the ACL graft. Cryotherapy is applied to the surgical site. On postoperative Day 3, the patient starts knee range-of-motion exercise using a continuous passive motion device. Knee range-of-motion training is provided to attain 90 degrees of flexion by the end of Week 2 and 120 degrees of flexion by the end of Week 3. The patient is provided with a soft brace for ACL injuries, and is allowed to walk with one third partial weight bearing at postoperative 2 weeks, a half partial weight bearing at postoperative 3 weeks. Patients were allowed to walk with full weight bearing between postoperative Weeks 4 and 6. To prevent delayed bone union, patients treated with this technique are currently advised to refrain from walking more than 3000 steps per day for the first 2 months postoperatively.

## Results

**Fig. 9:**
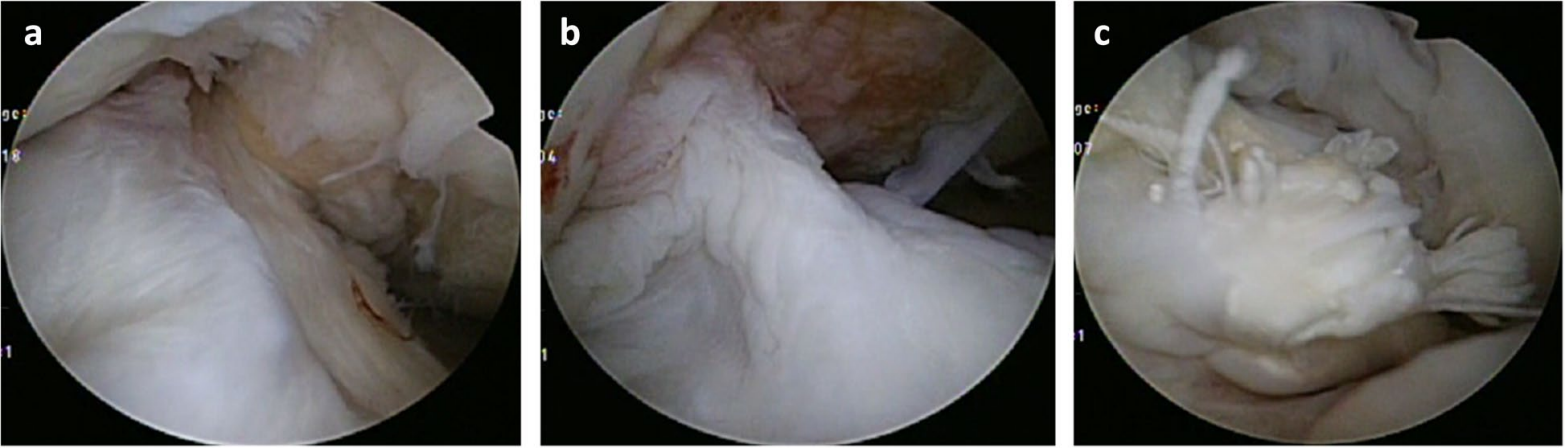
**a** Arthroscopic findings after ACL reconstruction. a Good tension and volume of the graft, 13 knees (76.4%). **b** Looseness or tear of anterior fiber of the graft, 3 knees (17.6%). **c** A cyclops of the graft, 1 knee (5.9%)

Preoperatively, these patients had a mean femorotibial angle (FTA) of 183.8° (standard deviation [SD] 3.4°, range 180°–190°), medial proximal tibial axis (MPTA) of 81.6° (SD 3.0°, range 77–87°), mean side-to-side difference in anterior–posterior knee laxity (assessed using a KT-1000 arthrometer, Medmetric, San Diego, CA) of 4.1 mm (SD 1.3 mm, range 3–7 mm), mean preoperative JOA score of 65.0 (SD 13.5), and mean Lysholm score of 47.2 (SD 16.2). Preoperative pivot shift was positive in 18 knees, negative in one knee. Mean postoperative follow-up period was 31 months (range 24–49 months). After surgery, the mean JOA-OA score, Lysholm score, and side-to-side difference in KT-1000 measurements improved to 93.1 ± 6.0 (*P* < 0.00001), 94.2 ± 5.8 (*P* < 0.00001), and -0.2 ± 0.8 mm (*P* < 0.00001), respectively (Table 1). Postoperative X-ray imaging showed that the mean standing FTA decreased to 168.0 ± 3.3 (*P* < 0.00001), and the mean posterior tibial slope angle decreased to 5.0 ± 3.6° (from 6.9 ± 2.6° preoperatively; *P* = 0.023) (Table 2). The pre- and postoperative mean Insall-Salvati ratio of the patella was 1.0 ± 0.2 and 0.9 ± 0.2 (*P* = 0.03), respectively, and the pre- and postoperative mean Caton-Deschamps (C-D) index was 0.9 ± 0.2and 0.8 ± 0.2 (*P* = 0.008), respectively, indicating a mild decrease in the height of the patella after surgery (Table 2). Arthroscopy was performed on 17 knees at the time of plate removal at a mean of 16 months after surgery, and no ACL complete graft rupture was found. Arthroscopic findings of ACL graft in 13 knees (76.4%) were successful with good tension using a probe and good volume of arthroscopic finding (Fig. 9-a). Three knees (17.6%) of looseness or tear of the anterior fibers of the graft (Fig. 9-b), one knee (5.6%) of a cyclops lesion were observed (Fig. 9-c). These results indicated substantial improvements in knee stability and pain management. However, one patient showed non-union at the osteotomy site and underwent additional surgery.

**Table 1:** Clinical results of the patients between pre and post operation

	**Pre op**	**Post op**	**T-test**
JOA-OA Score (points)	65.0 ± 13.5	93.1 ± 6.0	*P* < 0.00001
Lysholm Score (points)	47.2 ± 16.2	94.2 ± 5.8	*P* < 0.00001
KT-1000: side to side difference (mm)	4.1 ± 1.3 (3–7)	- 0.2 ± 0.8 (-1 ~ 2)	*P* < 0.00001

**Table 2:** Radiographic findings the patients between pre and post operation

	**Pre op**	**Post op**	**T-test**
FTA	183.8 ± 3.4° (180 ~ 190°)	168.0 ± 3.3°(164 ~ 174°)	*P* < 0.00001
MPTA	81.6 ± 3.0°	94.7 ± 3.6°	*P* < 0.00001
Posterior slope angle	6.9 ± 2.6°	5.0 ± 3.6°	*P* = 0.024
Insall-Salvati ratio	1.0 ± 0.2	0.94 ± 0.2	*P* = 0.033
Caton-Deschamps (C-D) index	0.94 ± 0.2	0.8 ± 0.2	*P *= 0.008

## Discussion

The most important finding of this study was followings. The combination of dome-shaped HTO with all-inside ACL generally alleviated pain and instability of the knee. Radiographic imaging showed that the posterior tibial slope was decreased while the height of the patella was mildly reduced. Dome-shaped HTO allows for a relatively high degree of valgus correction and decreases the steep posterior tibial slope that causes excessive load on the ACL. Therefore, its use in combination with ACL reconstruction seems to be effective.

Total knee arthroplasty is indicated for patients with knee osteoarthritis and instability due to ligament deficiencies, particularly when these individuals are elderly and have severe varus deformities [9]. However, careful consideration should be given to surgical options for patients who are engaged in heavy labor, who wish to enjoy running, mountain climbing, or other sports, or who are under the age 70 and wish to maintain a physically active or floor-sitting lifestyle [4, 8].

Recently, increasing numbers of studies reported the clinical results of the combination of HTO and ACL reconstruction [4, 5]. While most of these results were satisfactory, in some cases they were poor [13, 20]. According to recent statistics, opening-wedge HTO is preferred over closing-wedge HTO [3, 32, 37]. The decision to perform HTO alone or in combination with ACL reconstruction involves consideration of patient demographics, symptoms and ligaments involved [8, 34]. A combined HTO and ACL reconstruction showed excellent results in a long-term follow-up in young patients even in severe osteoarthritis [26], but uncertain return to high level sport [24].

Opening-wedge HTO poses the risk of increasing the posterior tibial slope [28, 35], which results in excess strain on the ACL [15]. However, there are techniques how to avoid an increase of the posterior tibial slope during Opening-wedge HTO.

In contrast, dome-shaped HTO has a low risk of increasing the posterior tibial slope, although it is technically demanding to cut the tibial cortex along a steep curve. However, percutaneous drilling method is easy and obtain the accurate dome-shaped osteotomy [29]. In this series, average of posterior tibial slope was slightly decreased. In this dome shaped osteotomy, two trapezoidal HA blocks are inserted medially, one medial and one posterior medial. Since the medial front is steep and there is no space for installation, only the cancellous bone tip is inserted. Therefore, it is thought that the posterior slope angle decreased.

Patellar height by I-S ratio was decreased after opening-wedge osteotomy by more than 20% in 28% of those patients, not changed after closing-wedge osteotomy [1]. Regarding the patellar height, this series showed the slightly lower than that of before surgery. However, the average decrease of I-S ratio was only 6.3%, by more than 20% in 3 knees (6.3%) of this series.

An inverted V-shaped high tibial osteotomy (IV HTO) [16] did not change posterior tibial slope, the patellar height, however, including the technical difficulty of performing a precise inverted V-shaped osteotomy [27]. In contrast, this method is less technical demanding than that of IV HTO, because precise preoperative planning is not necessary, and correction angle is able to change during intraoperative correction under a fluoroscopic control. However, IV HTO has advantage of no change of posterior tibial slope and the patellar height [17], The indication and clinical results of IV HTO, opening-wedge HTO, closing-wedge HTO, and dome shaped osteotomy should be discussed.

ACL reconstruction techniques can be categorized into two types regarding tibial and femoral tunnel placement such as independent and dependent. With the independent approach, tibial and femoral tunnels are created independently of each other. With the dependent approach, the femoral tunnel is created in a manner that is dependent on the tibial tunnel [30], or vice versa [31]. Currently, the independent approach is the technique of choice among many surgeons [6, 10, 23]. In contrast, studies report that modified transtibial approaches by dependent procedures achieve successful outcome [19, 36]. Based on its excellent short-term results for knee stability and pain management, our dependent transfemoral ACL reconstruction technique was applied to the case series reported here.

With the all-inside ACL reconstruction employed here, there were very low risks of HTO fixation screws interfering with tibial drilling since tibial sockets were created from inside the joint cavity. Our combination surgery was relatively easy to perform, and its results are promising. Patients aged 70 years or younger, those who had knee osteoarthritis complicated with ACL deficiencies, and those who wished to engage in full sporting activities or live a floor-sitting lifestyle were treated with a combination of dome-shaped HTO and all-inside transfemoral ACL reconstruction.

There was a small number of cases of this procedure. We must perform this case series furthermore, evaluate the knee stability such as KT-1000 measurement and usefulness and the indication of this procedure.

## Conclusions

Dome-shaped HTO allows for a relatively high degree of varus correction and decreases the steep posterior tibial slope that causes excessive load on the ACL. Moreover, it facilitated tibial socket placement and graft fixation in a minimally invasive manner. With all-inside ACL reconstruction, there was a low risk of HTO fixation screws interfering with tibial drilling since tibial sockets were created from inside the joint cavity.
